# A novel visualization approach for network meta-analysis: The plate plot and the nmaplateplot R package

**DOI:** 10.1017/rsm.2026.10088

**Published:** 2026-04-07

**Authors:** Yanan Ren, Zhenxun Wang, Lifeng Lin, Shanshan Zhao, Haitao Chu

**Affiliations:** 1 Medtronic Inc, Minneapolis, MN, USA; 2 Amgen Inc, Thousand Oaks, California, USA; 3 Department of Epidemiology and Biostatistics, The University of Arizona Mel and Enid Zuckerman College of Public Health, Tucson, Arizona, USA; 4 National Institute of Environmental Health Sciences, Research Triangle Park, North Carolina, USA; 5 Division of Biostatistics and Health Data Science, University of Minnesota, Minneapolis, Minnesota, USA; 6 Pfizer Inc, New York, New York, USA

**Keywords:** network meta-analysis, nmaplateplot, plate plot, visualization tool

## Abstract

Network meta-analysis (NMA) provides a powerful framework for synthesizing evidence across multiple interventions, accommodating both direct and indirect comparisons. However, effectively visualizing the complex, multidimensional results, such as effect magnitudes, uncertainty, *p*-values, and treatment rankings, remains a significant challenge. Outputs such as relative treatment effects, uncertainty, statistical significance, and treatment rankings are often reported separately, making it difficult for researchers and stakeholders to synthesize findings efficiently. We introduce *plate plot*, an innovative approach for visualizing key outcomes from NMA in a single, compact format. It enables simultaneous display of point estimates, confidence or credible intervals, significance levels, and surface under the cumulative ranking curve values, thereby facilitating clearer interpretation and communication of NMA findings. Using an example dataset, we demonstrate how the *plate plot* displays multiple relevant metrics to compare the efficacy and acceptability of various antidepressant interventions in a single, intuitive plot. The *plate plot*, generated effortlessly via the open-source *nmaplateplot* R package, enables users to generate customizable, publication-ready graphics with minimal programming. This tool enhances the ability to holistically evaluate and interpret complex comparative effectiveness data, supporting better-informed decision-making in research and clinical practice.

## Highlights

### What is already known?

- NMA enables the comparison of multiple interventions by synthesizing both direct and indirect evidence, offering a robust statistical framework for comparative effectiveness research.
- Effectively visualizing the complex, multidimensional results of NMA, such as effect magnitudes, uncertainty, *p*-values, and treatment rankings, is a significant challenge.

### What is new?

- The plate plot is a compact, customizable visualization tool that addresses this challenge by enabling the simultaneous display of point estimates, confidence intervals, significance levels, and surface under the cumulative ranking curve (SUCRA) values. It is implemented through the open-source nmaplateplot R package, allowing users to generate publication-ready figures with minimal coding.
- Using an example dataset, we demonstrate how the plate plot displays multiple relevant metrics to compare the efficacy and acceptability of various antidepressant interventions in a single, intuitive plot.

### Potential impact for RSM readers

- The plate plot enhances the ability to holistically evaluate and interpret complex comparative effectiveness data, supporting better-informed decision-making in research and clinical practice.
- More broadly, this work illustrates the value of purposeful, user-oriented visualization in advancing the utility and communication of quantitative synthesis results.

## 1 Introduction

In evidence-based medicine, systematic reviews and meta-analyses are widely used to synthesize evidence from multiple studies, providing meaningful insights to guide clinical and policy decision-making processes.^1^ ^,^ ^2^ Network meta-analysis (NMA), unlike conventional pairwise meta-analysis, which can only incorporate direct comparisons between interventions, enables the integration of both direct and indirect evidence. This allows for a comprehensive evaluation of multiple competing treatments with respect to efficacy and acceptability.^3^ ^–^ ^9^ Despite its advantages, the multidimensional nature of NMA outputs poses challenges for effective communication and interpretation of results.^10^ ^–^ ^12^

As a result, the development of visualization tools specifically tailored for NMA has emerged as a critical area of methodological research. These tools aim to facilitate the exploration, interpretation, and dissemination of complex NMA results.^13^ For instance, Veroniki et al. introduced the rank-heat plot, which displays treatment rankings based on SUCRAs across multiple outcomes to help identify the most favorable interventions.^14^ Seo et al. proposed the Kilim plot, a graphical representation emphasizing the absolute treatment effects across outcomes, with color-coded indicators of statistical significance.^15^ More recently, Nevill et al. enhanced the interactive web-based platform MetaInsight, by adding new visualization tools such as the Litmus Rank-O-Gram and the Radial SUCRA plot, offering a more comprehensive view of NMA results.^16^

However, a gap remains in the availability of graphic tools that can succinctly display multiple types of comparative effect measures, including ratio-scale measures (e.g., odds ratios or risk ratios) and difference-scale measures (e.g., risk differences or standardized mean differences), along with statistical significance, and treatment rankings within a single compact visualization. Moreover, an ideal tool would also support comparisons across different analytical frameworks (e.g., frequentist versus Bayesian NMA) and clearly distinguish results from primary and sensitivity analyses.

The aim of this manuscript is to introduce a comprehensive and versatile graphical tool, the plate plot and the nmaplateplot R package. This tool can simultaneously display up to two types of outcomes—one in the upper diagonal and the other in the lower diagonal; moreover, the diagonals can also represent results from different statistical frameworks or analyses, for instance, contrasting Bayesian versus frequentist NMA, primary versus sensitivity analyses, assessments of consistency versus inconsistency, or comparing pairwise meta-analysis versus NMA. Its usage is illustrated by an example that integrates efficacy and acceptability outcomes of antidepressants, color-coded significance indicators, and SUCRA ranking in NMA into a unified visualization. We also demonstrate the tool’s flexibility through customization options that allow researchers to tailor the visual output to their specific study needs.

## 2 An illustrative example: Efficacy and acceptability of 12 antidepressants

To demonstrate the utility of the plate plot, we applied it to the Bayesian NMA and pairwise meta-analysis results generated from the raw data of a previously published NMA,^17^ which assessed the efficacy and acceptability of 12 new-generation antidepressants for the acute treatment of major depressive disorder. This NMA provided a comprehensive set of relative effect estimates and treatment rankings across multiple interventions.

As input, the *plateplot* function within the R package *nmaplateplot* requires a list of (at least) five data frames, each corresponding to one of the key components of the NMA results: point estimates, lower and upper bounds of confidence or credible intervals, *p*-values (or Bayesian posterior probabilities for Bayesian analyses, hereafter referred to as *p*-values for simplicity), and treatment identifiers (e.g., abbreviated treatment names). The example input data frames are provided in Supplementary Appendix A, and the corresponding plate plot outputs are shown in Supplementary Appendix B. These examples demonstrate both supported output formats—the row–column (rc) and the upper-left–lower-right (ullr) layout—and explain how each layout organizes pairwise treatment comparisons. Guidance is also provided for interpreting the plate plot, including how individual cells correspond to treatment contrasts typically displayed in a forest plot (e.g., effects relative to the VEN treatment).

### 2.1 The structure of the plate plot

**Figure 1:**
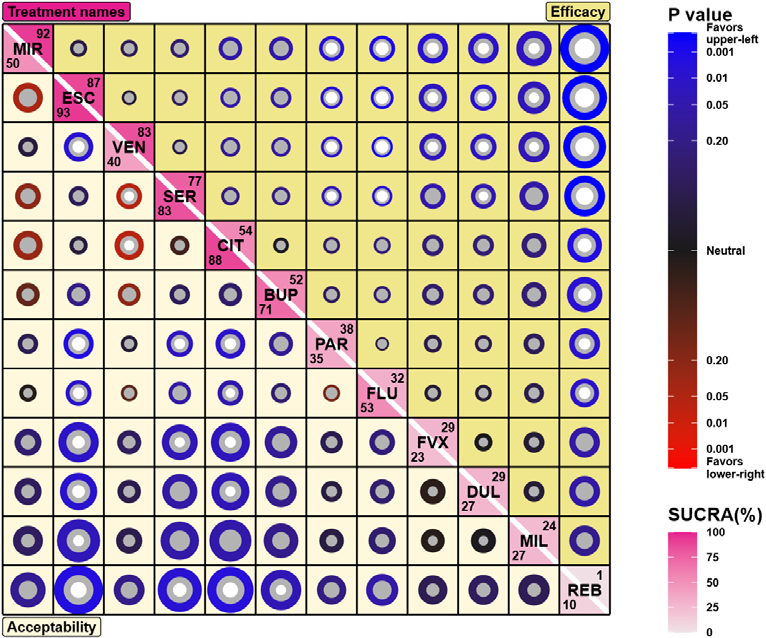
Efficacy and acceptability of 12 antidepressants shown in a plate plot. Treatment identifiers: MIR, mirtazapine; ESC, escitalopram; VEN, venlafaxine; SER, sertraline; CIT, citalopram; BUP, bupropion; PAR, paroxetine; FLU, fluoxetine; FVX, fluvoxamine; DUL, duloxetine; MIL, milnacipran; REB, reboxetine. Treatments are ordered according to SUCRA ranking for efficacy, with the highest-ranking treatments positioned in the top left and the lowest-ranking in the bottom right. Circles indicate the point and interval estimates: the gray circle marks the point estimate, while the colored outer circle (blue favors upper-left treatment, and red favors lower-right) shows the upper or lower bound of the confidence interval, depending on the direction of the effect. When results are statistically significant (p < 0.05), a white inner circle is added to denote the opposite bound of the interval. The color intensity corresponds to p-value thresholds, as indicated in the legend.

Figure 1 presents a plate plot, generated by the *plateplot* function in the R package *nmaplateplot*, depicting the efficacy (in the upper triangular matrix with yellow background color) and acceptability (in the lower triangular matrix with light yellow background color) of 12 antidepressants.

Each off-diagonal cell represents a pairwise comparison between two treatments and contains either two (if *p*-value > 0.05) or three (for *p*-value < 0.05) concentric circles:

- The inner circle (white) appears only for statistically significant results and represents the lower bound of interval estimates if the effect is positive with a null value of zero (e.g., risk difference) or greater than one with a null value of one (e.g., risk ratio, odds ratio), or the upper bound if the effect is negative with a null value of zero (or less than one under a null value of one).
- The middle circle (gray) indicates the point estimate.
- The outer circle, color-coded by *p*-values, represents the upper bound of the interval estimate if the effect is positive with a null value of zero (or greater than one with a null value of one), or the lower bound if the effect is negative with a null value of zero (or less than one under a null value of one). The specific colors correspond to significance levels as shown in the accompanying color bar in the legend.

The radius of the circles is proportional to the magnitude of the effect size and confidence interval, allowing for a quick visual interpretation of effect size. For instance:

- A plate with blue/gray/white coloring in the cell at the intersection of the first row and twelfth column indicates that mirtazapine (MIR) is statistically significantly more effective than reboxetine (REB) in terms of response rate (efficacy).
- A plate with red/gray coloring in the cell at the intersection of the second row and first column indicates that MIR is less acceptable (i.e., associated with higher dropout rates) than escitalopram (ESC), although the result is not statistically significant.

Drug names are displayed along the diagonal of the plot. Within each diagonal cell, two numeric values represent the SUCRA (%) scores for efficacy and acceptability for the corresponding treatment. The background colors of the diagonal cells reflect the SUCRA values, consistent with the color scale shown in the legend.

### 2.2 Text-based display of network meta-analysis results

**Figure 2:**
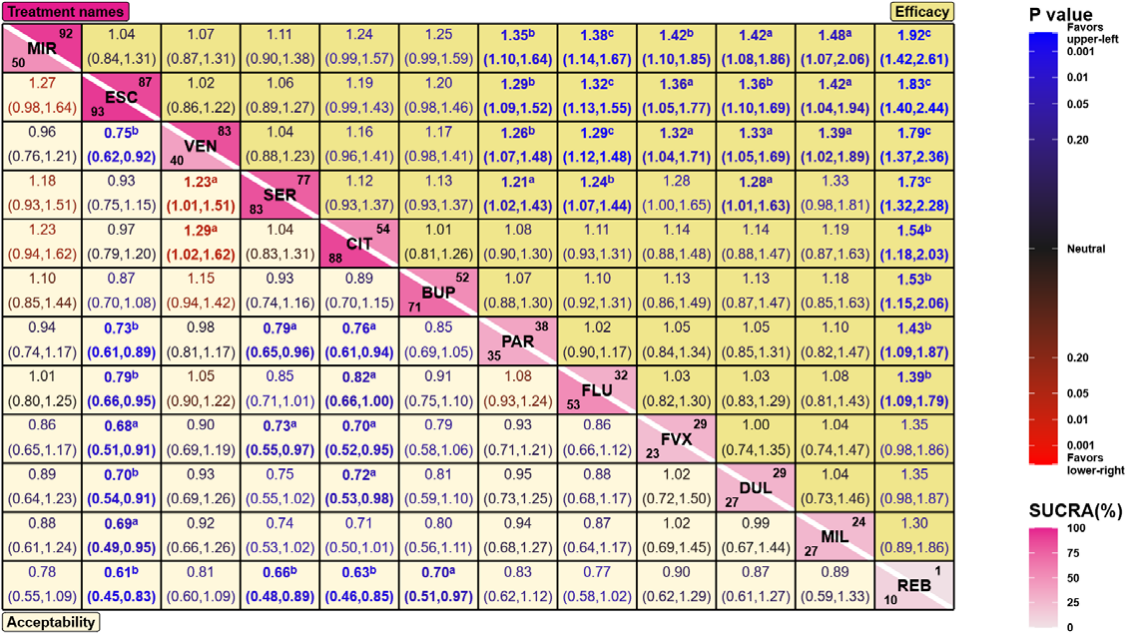
Efficacy and acceptability of 12 antidepressants presented as an enhanced league table with point and interval estimates, SUCRA ranking, and significance information. Treatment identifiers: MIR, mirtazapine; ESC, escitalopram; VEN, venlafaxine; SER, sertraline; CIT, citalopram; BUP, bupropion; PAR, paroxetine; FLU, fluoxetine; FVX, fluvoxamine; DUL, duloxetine; MIL, milnacipran; REB, reboxetine. Treatments are ordered according to SUCRA ranking for efficacy, with the highest-ranking treatments positioned in the top left and the lowest-ranking in the bottom right. Superscripts a, b, and c in the cells indicate p < 0.05, 0.01, and 0.001, respectively.

In addition to the graphical plate plot shown in Figure 1, the *plateplot* function also supports a text-based display of NMA results. By setting the option *design_method = text*, users can generate a matrix of pairwise comparisons where each cell directly reports the point estimate along with its 95% credible interval, as illustrated in Figure 2.

Each cell presents the odds ratio (OR) and its 95% credible interval for a given comparison. Results with *p*-values < 0.05 are shown in boldface for emphasis. Additionally, significance levels are indicated using superscript letters “a”, “b”, and “c” (as shown in Figure 2 and Figure C1 in Supplementary Appendix C, default in the package, example R code provided in Supplementary Appendix D) or traditional symbols “*”, “**”, and “***” (example R code provided in Supplementary Appendix E), corresponding to *p*-values of < 0.05, < 0.01, and < 0.001, respectively. Furthermore, the cell background color can be customized to reflect whether a comparison exceeds a user-defined threshold, allowing users to highlight clinically or statistically meaningful differences. Supplementary Appendix F provides an example demonstrating how to set these thresholds and apply the customized coloring, along with the corresponding R code.

The text color in each cell in Figure 2 corresponds to the *p*-value color bar on the left side of the plate plot for visual consistency. Blue text indicates that the results favor the treatment shown in the upper-left position, while red text indicates that they favor the lower-right treatment. For example: “1.92 (1.42, 2.61)” is displayed in blue in the cell at the intersection of the first row and twelfth column, indicating a preference for the upper-left treatment (MIR) over the lower-right (REB) in efficacy whereas “1.27 (0.98, 1.64)” is shown in red in the cell at the intersection of the second row and first column, showing the odds ratio of acceptability for MIR versus ESC, indicating ESC has better acceptability compared to MIR.

It is important to note that the input dataset for the *plateplot* function follows a row–column (rc) layout format, where relative effect measures (e.g., odds ratios) are calculated by comparing the odds of the treatment in the row against the treatment in the column. This layout simplifies data preparation.

However, by default, the *plateplot* function displays results using an upper-left versus lower-right (ullr) format, where each cell shows the relative effect (e.g., odds ratio) of the treatment in the upper-left corner compared to the treatment in the lower-right. To ensure that the orientation of the plot matches the row–column layout of the input data, users can set the argument option *transform_rc_ullr_boolean = FALSE*. Leaving this option at its default (TRUE) preserves the upper-left vs. lower-right display format.

### 2.3 Additional plot parameters

Beyond the two primary outcomes (efficacy and acceptability) illustrated in Figures 1 and 2, the *plateplot* function is flexible and capable of displaying a wide range of outcome types. Users can customize the visualization to depict other relative effect measures such as relative risk (RR) and RD. Figure C1 provides an example of this extended functionality, showing both the RR and RD of the efficacy of 12 antidepressant treatments. Depending on the type of outcomes being visualized, two key options must be specified to correctly interpret treatment comparisons: *lower_better* and *null_value_zero.*

The *lower_better* argument is a vector of two logical values, corresponding to the upper and lower diagonal parts of the plot. It indicates whether lower estimates represent better outcomes. For example, because lower dropout rates (i.e., higher acceptability) are preferable, this should be set to TRUE for acceptability outcomes. In contrast, for efficacy outcomes where higher effect sizes are favorable, this should be set to FALSE. Therefore, for a typical efficacy (upper triangle) vs. acceptability (lower triangle) plot, the setting would be *lower_better = c(FALSE, TRUE)*.

The *null_value_zero* argument specifies the neutral reference point for interpreting effect sizes. It also takes a vector of two logical values for the upper and lower diagonal parts, respectively. Set this to *TRUE* when the null value is zero (e.g., for risk difference or standardized mean difference) and to *FALSE* when the null value is 1 (e.g., for risk ratio or odds ratio). For instance, when plotting odds ratios for efficacy and risk differences for acceptability, the setting would be *null_value_zero = c(FALSE, TRUE)*.

### 2.4 Ordering treatments

By default, treatments in the plate plot are arranged according to their SUCRA values, with higher-ranking treatments positioned earlier. This automatic ordering facilitates interpretation when treatment ranking is the primary focus.

However, users have the flexibility to define a custom treatment order by specifying the *Order* argument. This argument accepts a user-defined vector that determines the sequence in which treatments appear along both axes of the plot. This feature is particularly useful when aligning treatments to match external references, clinical guidelines, or specific analytical priorities.

### 2.5 NMA results with missing outcome data

In practice, missing information in network evidence can arise not only from the absence of direct head-to-head studies but also from studies that do not report the outcome of interest for one or more treatment arms or from treatment comparisons that rely solely on indirect evidence because the requisite outcome data are unavailable.

**Figure 3:**
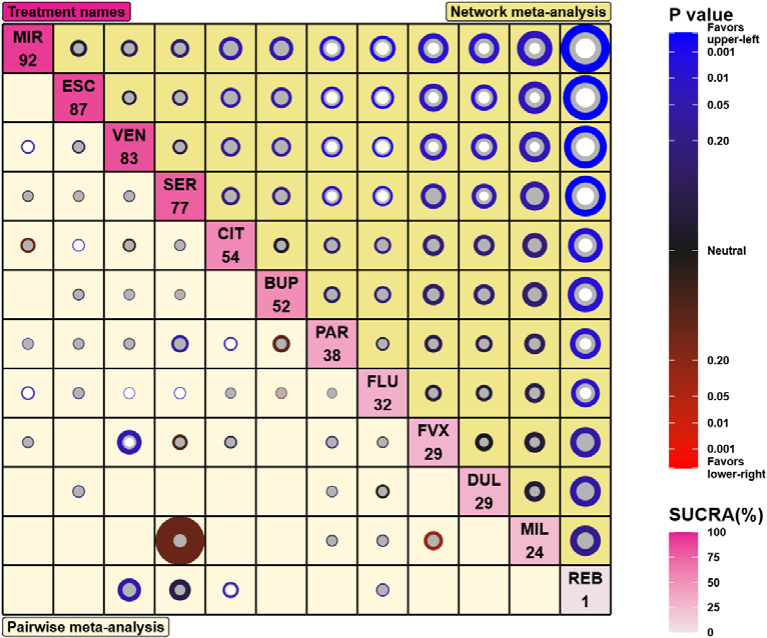
Comparing the efficacy of 12 antidepressants: network meta-analysis and pairwise meta-analysis. Missing values in pairwise meta-analysis were shown as blank cells. Treatment identifiers: MIR, mirtazapine; ESC, escitalopram; VEN, venlafaxine; SER, sertraline; CIT, citalopram; BUP, bupropion; PAR, paroxetine; FLU, fluoxetine; FVX, fluvoxamine; DUL, duloxetine; MIL, milnacipran; REB, reboxetine. Treatments are ordered according to SUCRA ranking for efficacy, with the highest-ranking treatments positioned in the top left and the lowest-ranking in the bottom right. Circles indicate the point and interval estimates: the gray circle marks the point estimate, while the colored outer circle (blue favors upper-left treatment, and red favors lower-right treatment) shows the upper or lower bound of the confidence interval, depending on the direction of the effect. When results are statistically significant (p < 0.05), a white inner circle is added to denote the opposite bound of the interval. The color intensity corresponds to p-value thresholds, as indicated in the legend.

In scenarios with no head-to-head comparison between two interventions, conducting a pairwise meta-analysis becomes infeasible due to the lack of direct evidence.^5^ The *nmaplateplot* package accommodates this limitation by enabling separate visualization of results from NMA and pairwise meta-analysis within the upper and lower triangular parts of the plot. While NMA synthesizes both direct and indirect evidence across multiple interventions, pairwise meta-analysis is restricted to direct comparisons available within individual studies. When no direct comparison exists for a treatment pair, the corresponding cell in the pairwise meta-analysis (typically the lower diagonal) is left blank, clearly indicating the absence of direct evidence. Figure 3 and Figure C2 illustrate this scenario, where numeric values for efficacy are shown only for treatment pairs with available direct comparisons in the pairwise meta-analysis.

The side-by-side display of network and pairwise estimates also facilitates an implicit evaluation of consistency within the evidence network. For instance, in Figure C2, the contrast between the upper-diagonal estimate (1.29, 95% CI 1.12–1.48) for VEN versus FLU and its lower-diagonal (1.36, 95% CI 1.14–1.62) counterpart is minimal, indicating good agreement between synthesized (direct and indirect) evidence and direct comparison. By contrast, for the SER versus MIL comparison, the upper-diagonal estimate (1.33, 95% CI 0.98–1.81) suggests a more favorable outcome for SER than the corresponding pairwise estimate (0.48, 95% CI 0.08–2.87), pointing to potential inconsistency and warranting further investigation.

## 3 Discussion

In this article, we introduce a novel visualization tool—the plate plot—implemented in the R package *nmaplateplot*, designed to present results from NMA involving multiple interventions in a concise and informative manner. This tool integrates multiple layers of information—such as ratio-scale effect size (e.g., odds ratios, risk ratios) and difference-scale effect size (e.g., risk differences, standardized mean differences), confidence intervals, statistical significance, and ranking metrics—within a single visual framework. The color scheme highlights statistical significance and SUCRA-based rankings, while the size of the circles (plates) visually represents the magnitude and precision of effect estimates. This enables both comprehensive and pairwise comparisons.

Beyond its visual appeal, the plate plot also provides numeric representations of effect sizes, confidence intervals, and SUCRA values directly within the graphic, functioning as an enhanced league table. Its high adaptability enhances usability across diverse research needs. For example, while treatments are ordered by SUCRA values by default, users can customize the order based on their own priorities. Additional features include the simultaneous display of ratio-scale and difference-scale outcomes (e.g., Figure C1), customizable circle size and color schemes, and handling of missing data, altogether enhancing the interpretability of complex NMA results. Importantly, the plate plot is intended to complement—not replace—existing visualizations commonly used in NMA. Forest plots remain essential for presenting precise estimates and interval widths, whereas the plate plot provides an efficient overview that helps authors quickly identify key patterns or contrasts warranting closer examination. Its league-table-style structure enables rapid qualitative assessment before consulting more detailed displays such as forest plots.

The plate plot is also well suited for comparing results across different analytical frameworks, such as Bayesian versus frequentist models (Supplementary Appendix G) or consistency versus inconsistency assumptions. Users can leverage the upper and lower triangular regions of the plot to contrast results from primary versus sensitivity analyses or across different modeling strategies. This feature facilitates deeper insights into agreement or divergence of model outputs under different scenarios, addressing the challenges highlighted in recent work on multivariate meta-regression and NMA modeling frameworks.^18^ In particular, inconsistency (disagreement between direct and indirect comparisons) remains a challenge in NMA. Modeling inconsistency through design-by-treatment interactions allows the total variability to be decomposed into heterogeneity and inconsistency components.^19^ The plate plot complements this evaluation by enabling visual comparison of consistency and inconsistency model outputs across its triangular regions. These contrasts help identify incoherent evidence loops and highlight where treatment effects diverge under different modeling assumptions, thereby improving transparency and confidence in the evidence synthesis.

Looking ahead, we aim to develop an interactive Shiny application to further enhance the accessibility and functionality of the plate plot. This platform would allow users to upload model outputs, configure visualization settings in real time, and export publication-quality graphics. This effort aligns with the broader movement in the evidence synthesis community toward interactive, web-based tools, as seen in platforms like MetaInsight,^20^ NMAstudio web app,^21^ IPDmada for diagnostic meta-analysis,^22^ RIMeta for reference interval estimation,^23^ and MA-cont for continuous outcome meta-analysis.^24^

Finally, tools like the plate plot support best practices in evidence reporting as advocated by guidelines such as the PRISMA extension for NMA.^25^ By summarizing multidimensional outcomes in a compact, customizable graphic, the plate plot contributes to clearer, more transparent reporting of intervention comparisons, model assumptions, and treatment rankings.

In conclusion, the plate plot offers a powerful and user-friendly solution to the challenges of visualizing complex NMA results. With its comprehensive features and flexibility, the tool empowers researchers to explore, interpret, and communicate synthesized evidence more effectively, ultimately supporting better-informed clinical and policy decisions.

## Supporting information

10.1017/rsm.2026.10088.sm001 Ren et al. supplementary material Ren et al. supplementary material

## Data Availability

The data that support the findings of this study are openly available in the R package “nmaplateplot” at https://cran.r-project.org/web/packages/nmaplateplot/index.html.

## Supplementary material

To view supplementary material for this article, please visit http://doi.org/10.1017/rsm.2026.10088.
