# Supplementary material for: A novel visualization approach for network meta-analysis: The plate plot and the nmaplateplot R package
Source: Res Synth Methods. 2026 Apr 7;17(5):1045–53. doi: 10.1017/rsm.2026.10088 (PMC13289567; doi:10.1017/rsm.2026.10088)
Supplement: Supplementary file 1 [file S175928792610088Xsup001.docx]

A novel visualization approach for network meta-analysis: the plate plot and the nmaplateplot R package

Appendices

**Appendix A: Example input dataset**

The input dataset must be a list containing at least 5 data frames named: Treatment_specific_values, Point_estimates, Interval_estimates_LB, Interval_estimates_UB, and Pvalues, which are listed in sequence below.

In the example, 12 treatment arms are labeled with Trt_ID (1–12), and abbreviated names are stored in the Trt_abbrv column. The rows and columns (V1–V12) of the estimate and p-value data frames correspond to the treatment order defined in Treatment_specific_values.

The dataset follows a row–column (rc) layout, where each cell represents the odds ratio for the row-defined treatment relative to the column-defined treatment. This dataset contains two outcomes, efficacy and acceptability, which are represented separately in the upper and lower diagonals of each input data frame, corresponding to the two diagonals in the plate plot.

For example:

a) *0.85 (0.71, 1.02)* in row 1, column 12 indicates the odd ratio of efficacy for BUP compared with VEN.
b) *1.15 (0.94, 1.42)* in row 12, column 1 indicates the odd ratio of acceptability for VEN compared with BUP.

Note: To preserve the row–column (rc) orientation of the input dataset in the plateplot output, set transform_rc_ullr_boolean = FALSE. By default, transform_rc_ullr_boolean = TRUE, which displays results in the upper-left–to–lower-right (ullr) layout. In this format, the odds ratio of acceptability in example b) is shown as the upper-left treatment (BUP) compared with the lower-right treatment (VEN), with an estimated value of 0.87 (0.71, 1.07).

**Appendix B: Example plateplot output and other visualizations for Appendix A**

The plateplot in row-column (rc) layout can be generated by the R code below:

Figure B1. Efficacy and acceptability of the 12 antidepressant treatments presented in a row-column layout. Treatment identifiers (in alphabetical order): BUP, bupropion; CIT, citalopram; DUL, duloxetine; ESC, escitalopram; FLU, fluoxetine; FVX, fluvoxamine; MIL, milnacipran; MIR, mirtazapine; PAR, paroxetine; REB, reboxetine; SER, sertraline; VEN, venlafaxine. Treatments are arranged alphabetically from the top left to the bottom right of the matrix. Cells with bold red values indicate that the treatment in the column performed significantly better than the treatment in the row, while bold blue values indicate that the treatment in the row performed significantly better than the treatment in the column.

The plateplot in upper left-lower right (ullr) layout can be generated by the R code below:

Figure B2. Efficacy and acceptability of the 12 antidepressant treatments presented in the upper left-lower right layout. Treatment identifiers (in alphabetical order): BUP, bupropion; CIT, citalopram; DUL, duloxetine; ESC, escitalopram; FLU, fluoxetine; FVX, fluvoxamine; MIL, milnacipran; MIR, mirtazapine; PAR, paroxetine; REB, reboxetine; SER, sertraline; VEN, venlafaxine. Treatments are arranged alphabetically from the top left to the bottom right of the matrix. Cells with bold red values indicate that the treatment in the lower right performed significantly better than the treatment in the upper left, while bold blue values indicate that the treatment in the upper left performed significantly better than the treatment in the lower right.

As an example, the last column (corresponding to treatment VEN) in Figure B2 illustrates how the efficacy estimates in this column can be represented using a forest plot (Figure B3). The 11 remaining treatments are compared against VEN, with point estimates and 95% confidence intervals displayed on the left and corresponding significance levels (indicated by color and bold formatting in the plate plot) shown on the right. Treatments FLU, PAR, and REB were statistically inferior to VEN at the *p* < 0.01 level, while DUL, FVX, and MIL were inferior at the *p* < 0.05 level.

The plate plot provides an efficient visualization of multiple pairwise comparisons among treatments, condensing information that would otherwise require 24 separate forest plots (12 for efficacy and 12 for acceptability). It can serve as a powerful exploratory tool to identify promising treatment comparison patterns, which can then be further examined and validated using detailed forest plots.

Figure B3. The last column (VEN) from the plate plot (Figure B2) is illustrated here as a forest plot. The 11 other treatments are compared with VEN, showing point estimates, 95% confidence intervals, and P-value.

**Appendix C: Supplementary Figures in the main document**

Figure C1. Risk ratio and risk difference of the 12 antidepressant treatments presented as an enhanced league table with point and interval estimates, SUCRA ranking, and significance information. Treatment identifiers: MIR, mirtazapine; ESC, escitalopram; VEN, venlafaxine; SER, sertraline; CIT, citalopram; BUP, bupropion; PAR, paroxetine; FLU, fluoxetine; FVX, fluvoxamine; DUL, duloxetine; MIL, milnacipran; REB, reboxetine. Treatments are ordered according to SUCRA ranking for efficacy, with the highest-ranking treatments positioned in the top left and the lowest-ranking in the bottom right.

Figure C2. Network meta-analysis (shown in the upper diagonal part with circles) versus pairwise meta-analysis (shown in the lower diagonal part with numbers) for comparing multiple treatments’ efficacy, missing values in pairwise meta-analysis were shown as blank cells. Treatment identifiers: MIR, mirtazapine; ESC, escitalopram; VEN, venlafaxine; SER, sertraline; CIT, citalopram; BUP, bupropion; PAR, paroxetine; FLU, fluoxetine; FVX, fluvoxamine; DUL, duloxetine; MIL, milnacipran; REB, reboxetine. Treatments are ordered according to SUCRA ranking for efficacy, with the highest-ranking treatments positioned in the top left and the lowest-ranking in the bottom right. Circles indicate the point and interval estimates: the grey circle marks the point estimate, while the colored outer circle (blue for positive, red for negative) shows the upper or lower bound of the confidence interval, depending on the direction of the effect. When results are statistically significant (p < 0.05), a white inner circle is added to denote the opposite bound of the interval. The color intensity corresponds to p-value thresholds, as indicated in the legend.

**Appendix D: Example R code for figures**

```
# Load packages
library(nmaplateplot)
library(svglite)
library(tidyverse)


# Figure 1
fig1 <- plateplot(ad12.eff.acc, design_method = c("circle", "circle"), upper_diagonal_name = "Efficacy", lower_diagonal_name = "Acceptability")

# Figure 2. The design_method option controls whether the upper and lower diagonal parts display "text" or "circle", respectively.
fig2 <- plateplot(ad12.eff.acc, design_method = c("text", "text"), upper_diagonal_name = "Efficacy", lower_diagonal_name = "Acceptability")

# Figure 3.
fig3 <- plateplot(ad12.pma.nma, design_method = c("circle", "circle"),
                  upper_diagonal_name = "Network meta-analysis",
                  lower_diagonal_name = "Pairwise meta-analysis",
                  transform_rc_ullr_boolean = TRUE,
                  null_value_zero = c(FALSE, FALSE),
                  lower_better = c(FALSE, FALSE))
# Figure C1
efig1 <- plateplot(ad12.rr.rd,
          null_value_zero = c(FALSE, TRUE), lower_better = c(FALSE, FALSE), design_method = c("text", "text"),
          upper_diagonal_name = "Efficacy: Risk ratio", lower_diagonal_name = "Efficacy: Risk difference")

# Change Treatment order
ad12.eff.acc$Treatment_specific_values$Order <- c(1:6,12:7)
efig2 <- plateplot(ad12.eff.acc, null_value_zero = c(FALSE, FALSE), lower_better = c(FALSE, TRUE), design_method = c("circle", "circle"), upper_diagonal_name = "Efficacy", lower_diagonal_name = "Acceptability")

# Figure C2
efig3 <- plateplot(ad12.pma.nma, design_method = c("text", "text"),
          upper_diagonal_name = "Network meta-analysis",
          lower_diagonal_name = "Pairwise meta-analysis",
          transform_rc_ullr_boolean = TRUE,
          null_value_zero = c(FALSE, FALSE),
          lower_better = c(FALSE, FALSE))

# Save plot
ggsave("fig1.svg", plot = fig1, device = "svg", width = 6.8, height = 6, dpi = 600)
ggsave("fig2.svg", plot = fig2, device = "svg", width = 10, height = 6, dpi = 600)
ggsave("fig3.svg", plot = fig3, device = "svg", width = 6.8, height = 6, dpi = 600)
ggsave("efig1.svg", plot = efig1, device = "svg", width = 10, height = 6, dpi = 600)
ggsave("efig2.svg", plot = efig2, device = "svg", width = 6.8, height = 6, dpi = 600)
ggsave("efig3.svg", plot = efig3, device = "svg", width = 11, height = 6, dpi = 600)
```

Note: To ensure optimal visualization and proportional scaling of elements, the plate plot dimensions should be adjusted according to the display type. For plate plots presenting **circles** within each cell, a near-square layout (width ≈ **6.8 inches**, height ≈ **6 inches**) is recommended to preserve the symmetry and relative spacing of graphical elements. For plate plots displaying **text** within each cell, a more **rectangular layout** (width ≈ **10 inches**, height ≈ **6 inches**) is preferred to accommodate label length.

**Appendix E: Example R code for indicating significance levels with “*”, “**”, “***”**

```
# Load R packages
library(nmaplateplot)
library(svglite)
library(tidyverse)

# Function to create p-value symbol
pval_to_symbol <- function(p) {
    if (p < 0.001) return(paste0("***"))
    else if (p < 0.01) return(paste0("**"))
    else if (p < 0.05) return(paste0("*"))
    else return(NA)
}


# Apply pval_to_symbol function to Pvalues data frame in example data
ad12.eff.acc$Symbol_indicators <- as.data.frame(apply(ad12.eff.acc$Pvalues, c(1, 2),
  pval_to_symbol))

# Example figure with *, **, *** to indicate significance level
plateplot(ad12.eff.acc,
          null_value_zero = c(FALSE, FALSE), lower_better = c(FALSE, TRUE),
          design_method = c("text", "text"),
          upper_diagonal_name = "Efficacy", lower_diagonal_name = "Acceptability")
```

**Appendix F: Example R code of customizing cell color to reflect the treatment comparison that exceeds a user-defined threshold**

```
library(nmaplateplot)
library(tidyverse)


ad12.eff.acc$Color <- as.data.frame(matrix(NA, nrow = 12, ncol = 12))
# setup upper-diagonal background
for(j in 2:12){
    for(i in 1:(j-1)){
        if(ad12.eff.acc$Point_estimates[i, j]>=1.2 | ad12.eff.acc$Point_estimates[i, j]<=(1/1.2)){
            ad12.eff.acc$Color[i, j] <- "khaki"
        }else{
            ad12.eff.acc$Color[i, j] <- "white"
        }
    }
}
# setup lower-diagonal background
for(i in 2:12){
    for(j in 1:(i-1)){
        if(ad12.eff.acc$Point_estimates[i, j]>=1.2 | ad12.eff.acc$Point_estimates[i, j]<=(1/1.2)){
            ad12.eff.acc$Color[i, j] <- "cornsilk"
        }else{
            ad12.eff.acc$Color[i, j] <- "white"
        }
    }
}
efig5 <- plateplot(ad12.eff.acc,
          null_value_zero = c(FALSE, FALSE), lower_better = c(FALSE, TRUE),
          design_method = c("text", "text"),
          upper_diagonal_name = "Efficacy", lower_diagonal_name = "Acceptability")

ggsave("efig5_threshold.svg", plot = efig5, device = "svg", width = 11, height = 6, dpi = 600)
```

Figure F1. Efficacy and acceptability of 12 antidepressants presented as an enhanced league table with point and interval estimates, SUCRA ranking, and significance information. Treatment identifiers: MIR, mirtazapine; ESC, escitalopram; VEN, venlafaxine; SER, sertraline; CIT, citalopram; BUP, bupropion; PAR, paroxetine; FLU, fluoxetine; FVX, fluvoxamine; DUL, duloxetine; MIL, milnacipran; REB, reboxetine. Treatments are ordered according to SUCRA ranking for efficacy, with the highest-ranking treatments positioned in the top left and the lowest-ranking in the bottom right. Superscripts a, b, and c in the cells indicate p < 0.05, 0.01, and 0.001, respectively. The cell coloring reflects whether a treatment comparison exceeds a user-defined threshold (e.g., ≥1.2 or ≤1/1.2). Comparisons that do not exceed the threshold appear in white; those that do may be highlighted in “khaki” in the upper diagonal or “cornsilk” in the lower diagonal. **Both the threshold value and the cell colors are fully customizable by the user.**

**Appendix G: Example R code of comparing frequentist NMA vs Bayesian NMA from the packages netmeta and gemtc, respectively**

```
library(gemtc)
library(netmeta)
library(nmaplateplot)
library(dplyr)

### clean the data
data("Dogliotti2014")
cleandata <- Dogliotti2014
cleandata <- cleandata %>% mutate(
  treatment = case_when(
    treatment == "Placebo/Control" ~ "Placebo_Control",
    treatment == "Aspirin+Clopidogtrel" ~ "Aspirin_Clopidogtrel",
    treatment == "Dabigatran 110mg" ~ "Dabigatran_110mg",
    treatment == "Dabigatran 150mg" ~ "Dabigatran_150mg",
    TRUE ~ treatment
  )
)


### frequentist NMA

pw2 <- pairwise(treat = treatment, n = total, event = stroke,
        studlab = study, data = cleandata, sm = "OR")

## ASPO study is excluded from the NMA due to zero events in both treatment groups
pw2 <- pw2[!is.na(pw2$TE),]

fnet <- netmeta(pw2, common=TRUE, ref="plac")
print(fnet)

# point estimate
exp(fnet$TE.common["VKAs", "Placebo_Control"])
# confidence intervals
exp(fnet$upper.common["VKAs", "Placebo_Control"])
exp(fnet$lower.common["VKAs", "Placebo_Control"])

# p value
fnet$pval.common["VKAs", "Placebo_Control"]

# sucra
set.seed(12)
fran <- rankogram(fnet)
fsucra <- netrank(fran)


### Bayesian NMA
bayesdata <- cleandata
bayesdata$id <- NULL
colnames(bayesdata) <- c("study", "treatment", "responders", "sampleSize")
bayesdata <- bayesdata %>% mutate(
  study = as.factor(study),
  treatment = as.factor(treatment)
) %>% distinct(study, treatment, .keep_all=TRUE)

network.orr <- mtc.network(data.ab=bayesdata)

bayesmodel.orr_fixed <- mtc.model(network.orr, likelihood = "binom", link = "logit", linearModel = "fixed",
                         re.prior.sd = 100, n.chain = 4)
mcmc.orr_fixed <- mtc.run(bayesmodel.orr_fixed, n.adapt = 10000, n.iter = 100000, thin = 10)


summary(mcmc.orr_fixed )
relative_effect <- relative.effect(mcmc.orr_fixed, t1="Placebo_Control", t2="VKAs")
sum_relative_effect <- summary(relative.effect(mcmc.orr_fixed, t1="Placebo_Control", t2="VKAs"))

bayes_OR <- exp(sum_relative_effect$summaries$quantiles)

# Bayesian P-value
p_row <- c(relative_effect$samples[[1]], relative_effect$samples[[2]], relative_effect$samples[[3]],
           relative_effect$samples[[4]])
sum(p_row > 0)/length(p_row)
# point estimate
bayes_OR[3]
# 95% CI
bayes_OR[1]
bayes_OR[5]
# sucra
bayes_ranks <- rank.probability(mcmc.orr_fixed, preferredDirection = -1)
bscura <- sucra(bayes_ranks)
### construct nmaplateplot dataset

# assign treatment ID.
ntrt <- 8
Treatment_specific_values <- data.frame(1:ntrt, as.character(bayesmodel.orr_fixed$network$treatments[,"id"]))
colnames(Treatment_specific_values) <- c("Trt_ID", "Trt_abbrv")

Point_estimates <-  as.data.frame(matrix(0, ncol = ntrt, nrow = ntrt))
Interval_estimates_LB <- as.data.frame(matrix(0, ncol = ntrt, nrow = ntrt))
Interval_estimates_UB <- as.data.frame(matrix(0, ncol = ntrt, nrow = ntrt))
Pvalues <- as.data.frame(matrix(0, ncol = ntrt, nrow = ntrt))

freq_vs_bayes <- list(Treatment_specific_values= Treatment_specific_values, Point_estimates = Point_estimates,
                      Interval_estimates_LB = Interval_estimates_LB, Interval_estimates_UB = Interval_estimates_UB,
                      Pvalues = Pvalues)

# we put Bayesian results in lower diagonal while Frequentist results in upper diagonal
# The dataset you input should follow row-column (rc) layout,
# which means odds ratio in each cell should stand for odds in the row defined treatment
# compared with odds in the column defined treatment.
for(i in 2:ntrt){
  for(j in 1:(i-1)){
    print(i)
    print(j)
    # lower diagonal: Bayesian results
    trt1 <- Treatment_specific_values[j, "Trt_abbrv"]
    trt2 <- Treatment_specific_values[i, "Trt_abbrv"]
    relative_effect <- relative.effect(mcmc.orr_fixed, t1=trt1, t2=trt2)
    sum_relative_effect <- summary(relative.effect(mcmc.orr_fixed, t1=trt1, t2=trt2))
    bayes_OR <- exp(sum_relative_effect$summaries$quantiles)
    p_row <- c(relative_effect$samples[[1]], relative_effect$samples[[2]], relative_effect$samples[[3]],
               relative_effect$samples[[4]])

    freq_vs_bayes$Point_estimates[i, j] <- as.numeric(bayes_OR[3])
    freq_vs_bayes$Interval_estimates_LB[i, j] <- as.numeric(bayes_OR[1])
    freq_vs_bayes$Interval_estimates_UB[i, j] <- as.numeric(bayes_OR[5])
    freq_vs_bayes$Pvalues[i, j] <- 1-abs(1-2*(sum(p_row > 0)/length(p_row)))

    # upper diagonal: Frequentist
    freq_vs_bayes$Point_estimates[j, i] <- exp(fnet$TE.common[trt1, trt2])
    freq_vs_bayes$Interval_estimates_LB[j, i] <- exp(fnet$lower.common[trt1, trt2])
    freq_vs_bayes$Interval_estimates_UB[j, i] <- exp(fnet$upper.common[trt1, trt2])
    freq_vs_bayes$Pvalues[j, i] <- fnet$pval.common[trt1, trt2]

  }
}
# add sucra values
freq_vs_bayes$Treatment_specific_values$Value_Lower <- bscura
freq_vs_bayes$Treatment_specific_values$Value_Upper <- fsucra$ranking.common
freq_vs_bayes$Treatment_specific_values$Trt_abbrv <- c("Apixaban", "Aspirin", "Asp_Clopidogtrel","Dab110", "Dab150",
                                                       "Placebo","Rivaroxaban","VKAs" )
# display the dataset
freq_vs_bayes$Point_estimates
freq_vs_bayes$Interval_estimates_LB
freq_vs_bayes$Interval_estimates_UB
freq_vs_bayes$Pvalues
freq_vs_bayes$Treatment_specific_values


#### construct the plate plot
plot_p <- plateplot(freq_vs_bayes, design_method = c("circle", "circle"), max_substring = 7,
                  transform_rc_ullr_boolean = TRUE,
                  null_value_zero = c(FALSE, FALSE), lower_better = c(TRUE, TRUE),
                  upper_diagonal_name = "Netmeta", lower_diagonal_name = "Gemtc")
print(plot_p)

plot_t <- plateplot(freq_vs_bayes, design_method = c("text", "text"), max_substring = 7,
                    transform_rc_ullr_boolean = TRUE,
                    null_value_zero = c(FALSE, FALSE), lower_better = c(TRUE, TRUE),
                    upper_diagonal_name = "Netmeta", lower_diagonal_name = "Gemtc")
print(plot_t)
```

Figure G1. Frequntist (Netmeta) and Bayesian (Gemtc) network meta-analysis of 8 treatments for prevention of stroke, embolism and mortality in patients with atrial fibrillation shown in a nmaplate plot. Treatment identifiers: ASA, Aspirin; Asp_Clo, ASA plus clopidogrel; VKAs, vitamin K antagonists; Dab110, dabigatran 110 mg; Dab150, dabigatran 150 mg; Rivarox, rivaroxaban; Apixaba, apixaban; Placebo, placebo/control. Treatments are ordered according to SUCRA ranking for efficacy, with the highest-ranking treatments positioned in the top left and the lowest-ranking in the bottom right. Circles indicate the point and interval estimates: the grey circle marks the point estimate, while the colored outer circle (blue favors upper-left treatment, red favors lower-right treatment) shows the upper or lower bound of the confidence interval, depending on the direction of the effect. When results are statistically significant (p < 0.05), a white inner circle is added to denote the opposite bound of the interval. The color intensity corresponds to p-value thresholds, as indicated in the legend.

Figure G2. Frequntist (netmeta) and Bayesian (gemtc) network meta-analysis of 8 treatments for prevention of stroke, embolism and mortality in patients with atrial fibrillation presented as an enhanced league table with point and interval estimates, SUCRA ranking, and significance information. Treatment identifiers: ASA, Aspirin; Asp_Clo, ASA plus clopidogrel; VKAs, vitamin K antagonists; Dab110, dabigatran 110 mg; Dab150, dabigatran 150 mg; Rivarox, rivaroxaban; Apixaba, apixaban; Placebo, placebo/control. Treatments are ordered according to SUCRA ranking for efficacy, with the highest-ranking treatments positioned in the top left and the lowest-ranking in the bottom right.

In the network meta-analysis presented in the plateplot table, the treatment located in the upper-left position (e.g., dabigatran 150 mg) demonstrated the greater reduction in stroke risk relative to treatment located in the lower-right position (.e.g., placebo or control). Estimates were consistent across Bayesian and frequentist frameworks. In the Bayesian network meta-analysis conducted with **gemtc**, dabigatran 150 mg showed a substantially lower odds of stroke compared with placebo (OR≈0.26; 95% CrI, 0.10–0.36). The corresponding frequentist analysis performed using **netmeta** yielded similar results (OR≈0.27; 95% CI, 0.19–0.37), demonstrating strong concordance in both magnitude and direction of effect across methods. This alignment between analytic approaches reinforces the robustness of the comparative efficacy estimates and supports the conclusion that dabigatran 150 mg provides the most favorable stroke prevention within the network.
